# Open lung approach associated with high-frequency oscillatory or low tidal volume mechanical ventilation improves respiratory function and minimizes lung injury in healthy and injured rats

**DOI:** 10.1186/cc9291

**Published:** 2010-10-14

**Authors:** Joerg Krebs, Paolo Pelosi, Charalambos Tsagogiorgas, Liesa Zoeller, Patricia RM Rocco, Benito Yard, Thomas Luecke

**Affiliations:** 1 Department of Anaesthesiology and Critical Care Medicine, University Hospital Mannheim, Faculty of Medicine, University of Heidelberg, Theodor-Kutzer Ufer, 1-3, 68165 Mannheim, Germany; 2 Department of Ambient, Health and Safety, University of Insubria, Sevizio di Anesthesia B, Ospedale di Circolo e Fondazione Macchi viale Borri 57, 21100 Varese, Italy; 3 Laboratory of Pulmonary Investigation, Carlos Chagas Filho Biophysics Institute, Federal University of Rio de Janeiro, Av. Carlos Chagas Filho, s/n, Rio de Janeiro, 21949-902, Brazil; 4 Department of Internal Medicine V University Hospital Mannheim, Faculty of Medicine, University of Heidelberg, Mannheim, Germany, Theodor-Kutzer Ufer 1-3, 68165 Mannheim, Germany

## Abstract

**Introduction:**

To test the hypothesis that open lung (OL) ventilatory strategies using high-frequency oscillatory ventilation (HFOV) or controlled mechanical ventilation (CMV) compared to CMV with lower positive end-expiratory pressure (PEEP) improve respiratory function while minimizing lung injury as well as systemic inflammation, a prospective randomized study was performed at a university animal laboratory using three different lung conditions.

**Methods:**

Seventy-eight adult male Wistar rats were randomly assigned to three groups: (1) uninjured (UI), (2) saline washout (SW), and (3) intraperitoneal/intravenous *Escherichia coli *lipopolysaccharide (LPS)-induced lung injury. Within each group, animals were further randomized to (1) OL with HFOV, (2) OL with CMV with "best" PEEP set according to the minimal static elastance of the respiratory system (BP-CMV), and (3) CMV with low PEEP (LP-CMV). They were then ventilated for 6 hours. HFOV was set with mean airway pressure (P_meanHFOV_) at 2 cm H_2_O above the mean airway pressure recorded at BP-CMV (P_meanBP-CMV_) following a recruitment manoeuvre. Six animals served as unventilated controls (C). Gas-exchange, respiratory system mechanics, lung histology, plasma cytokines, as well as cytokines and types I and III procollagen (PCI and PCIII) mRNA expression in lung tissue were measured.

**Results:**

We found that (1) in both SW and LPS, HFOV and BP-CMV improved gas exchange and mechanics with lower lung injury compared to LP-CMV, (2) in SW; HFOV yielded better oxygenation than BP-CMV; (3) in SW, interleukin (IL)-6 mRNA expression was lower during BP-CMV and HFOV compared to LP-CMV, while in LPS inflammatory response was independent of the ventilatory mode; and (4) PCIII mRNA expression decreased in all groups and ventilatory modes, with the decrease being highest in LPS.

**Conclusions:**

Open lung ventilatory strategies associated with HFOV or BP-CMV improved respiratory function and minimized lung injury compared to LP-CMV. Therefore, HFOV with Pmean_HFOV _set 2 cm H_2_O above the Pmean_BP-CMV _following a recruitment manoeuvre is as beneficial as BP-CMV.

## Introduction

Mechanical ventilation is lifesaving for patients with acute lung injury (ALI) and acute respiratory distress syndrome (ARDS). However, it can cause ventilator-induced lung injury through alveolar overdistension or opening and closing of atelectatic lung regions [1].

None of the current strategies to prevent mechanical ventilation injury in ALI/ARDS patients provides optimal protection. For example, the standard of care for controlled mechanical ventilation (CMV) in these patients to prevent lung and distal organ injury [2] limits tidal volume (V_T_) to 6 ml/kg predicted body weight and end-inspiratory plateau pressure (P_plat_) below 30 cm H_2_O. However, low V_T _may not completely prevent tidal hyperinflation [3], sometimes causing alveolar derecruitment [4]. An "open lung" (OL) ventilatory strategy based on recruitment manoeuvres (RMs) to open the lung and on decremental positive end-expiratory pressure (PEEP) titration to set the "best PEEP" to maintain the lung open [5] may result in systemic organ injury because high PEEP levels may cause excessive parenchymal stress and strain and have negative hemodynamic effects [6,7].

In turn, high-frequency oscillatory ventilation (HFOV) [8] is characterized by the rapid delivery of small V_T _of gas and the application of high mean airway pressures. These characteristics make HFOV conceptually attractive as an ideal lung-protective ventilatory model, since high mean airway pressure may prevent cyclical derecruitment of the lung, and the small V_T _limits alveolar overdistension. HFOV has been shown to improve respiratory function and reduce the lung inflammatory response in animal models [9]. However, it is unclear whether HFOV helps reduce mortality or comorbidities in infants [10] and adults [11] with ALI/ARDS. The adequate setting for mean airway pressure during HFOV is a matter of debate, with alternative approaches based on either a standard table of recommended mean airway pressure and oxygen concentration combinations or individual titration matching the oxygenation response of each patient [8]. Furthermore, it has been proposed that the pathophysiology of ALI/ARDS may differ depending on the type of insult [12], affecting the response to different ventilatory strategies [13,14]. Therefore, it may be of interest to assess the effects of predefined ventilatory approaches in widely differing lung conditions.

We hypothesized that (1) an open lung (OL) approach using HFOV (OL-HFOV) is more beneficial than OL-CMV or low PEEP CMV, and (2) these ventilatory strategies may be affected by the underlying lung condition. To investigate these hypotheses, we assessed the effects of three ventilatory strategies (1) OL-HFOV, (2) OL-CMV, and (3) low PEEP CMV in three experimental scenarios: without injury, following saline washout (SW) or lipopolysaccharide (LPS)-induced lung injury. The SW has been considered as an acute, direct lung injury model, severely compromising gas-exchange and lung mechanics, while the LPS model has been considered a more chronic, "sepsis-like" model of indirect lung injury. Therefore, this study did not aim to compare modes of mechanical ventilation between these ALI models, but to assess the effects of various ventilator strategy in each model.

## Materials and methods

The study was approved by the Institutional Review Board for the care of animal subjects (University of Heidelberg, Mannheim, Germany). All animals received humane care in compliance with the Principles of Laboratory Animal Care formulated by the National Society for Medical Research and the Guide for the Care and Use of Laboratory Animals prepared by the National Academy of Sciences, USA.

### Animal preparation and experimental protocol

A total of 78 specific pathogen-free male Wistar rats (450-500 g) housed in standard condition with food and water given *ad libitum *were anesthetized by intraperitoneal (IP) injection of ketamine hydrochloride (50 mg/kg) and xylazine (2 mg/kg), with additional anaesthesia administered as needed. The level of anaesthesia was assessed by pinching the paw and tail throughout the experiments. The femoral artery and both femoral veins were cannulated with polyethylene catheter tubing (PE-50; neoLab, Heidelberg, Germany). The arterial line was used for continuous monitoring of heart rate (HR), mean arterial pressure and to collect intermittent blood samples (100 μl) for blood-gas analysis (Cobas b121, Roche Diagnostics GmbH, Vienna, Austria). As soon as venous access was available, anaesthesia was maintained with intravenous ketamine via an infusion pump (Braun Perfusor Secura ft; B. Braun Melsungen AG, Melsungen, Germany) at an initial rate of 20 mg/kg/hr. This infusion rate was increased as needed to prevent spontaneous respiration after mechanical ventilation was established. The animals were tracheotomised, intubated with a 14-G polyethylene tube (Kliniject; KLINIKA Medical GmbH, Usingen, Germany) and mechanically ventilated with a neonatal respirator (Babylog 8000; Draeger, Luebeck, Germany) using a pressure-controlled mode with a PEEP of 2 cm H_2_O, inspiratory/expiratory ratio (I:E) of 1:1 and fraction of inspired oxygen (FiO_2_) of 0.5. This FiO_2 _level was used throughout the entire experimental period. End-inspiratory pressure (P_insp_) was adjusted to maintain a V_T _of 6 ml/kg body weight. A variable respiratory rate of 80-90 breaths/min was applied to maintain a PaCO_2 _value within physiological range. A catheter with a protected tip was inserted into the oesophagus for measurement of end-expiratory (P_es,exp_) and end-inspiratory (P_es,insp_) oesophageal pressure. The balloon catheter was first passed into the stomach and then withdrawn to measure P_es_. Proper balloon position was confirmed in all animals by observing an appropriate change in the pressure tracing as the balloon was withdrawn into the thorax (changes in pressure waveform, mean pressure and cardiac oscillation) as well as by observing a transient increase in pressure during a gentle compression of the abdomen as described previously [15].

Norepinephrine (Arterenol; Aventis Pharma Deutschland GmbH, Frankfurt am Main, Germany) was infused with an additional fluid bolus of balanced electrolyte solution (Deltajonin; Deltaselect GmbH, Munich, Germany) through the other venous line as needed to keep systolic blood pressure above 60 mmHg. The total volume of fluid administered was recorded. Body temperature was maintained between 37 °C and 38.5 °C with a heating pad. Paralyzing agents were not used. The depth of anaesthesia was similar in all animals, and a comparable amount of sedative and anaesthetic drugs were administered in all groups.

### Experimental protocol

**Figure 1:**
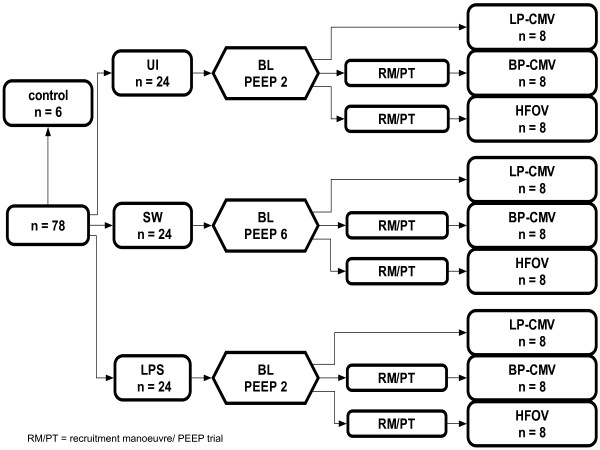
**Schematic flow chart of the study design**. UI, uninjured; SW, lung injury induced by saline washout; LPS, lung injury induced by intraperitoneal/intravenous *Escherichia coli *lipopolysaccharide; BL, baseline measurements; RM/PT, recruitment manoeuvre followed by decremental positive end-expiratory pressure (PEEP) trial; LP-CMV, controlled mechanical ventilation (CMV) with low PEEP; BP-CMV, controlled mechanical ventilation (CMV) with "best" PEEP; HFOV, high-frequency oscillatory ventilation.

A schematic flowchart of study design and the timeline representation of the procedure are shown in Figure 1. In the control (C) group (*n *= 6), animals were anaesthetized as described above and immediately killed by exsanguination via the vena cava. The remaining 72 animals were randomized into three groups (*n *= 24 each) and mechanically ventilated for 6 hours as follows: (1) uninjured (UI), (2) lung injury induced by saline washout (SW), and (3) lung injury induced by lipopolysaccharide (LPS; O55:B5) from *Escherichia coli *intraperitoneally/intravenously injected. Saline washout injury was induced as previously described [16]. Briefly, normal saline heated to body temperature (30 ml/kg body weight) was instilled via the endotracheal tube and removed via gravity drainage. After the first washout, the rats were alternately positioned on their left and right sides. After each lavage, P_insp _was readjusted to deliver V_T _of 6 ml/kg body weight. The procedure was repeated until a required P_insp _>22 cm H_2_O was obtained to maintain V_T _at 6 ml/kg body weight and PaO_2_/FiO_2 _below 100 mmHg. LPS injury was performed as a two-hit model by administering a single bolus of 1 mg/kg body weight intraperitoneally 24 hours prior to the experiment, followed by a constant intravenous infusion of LPS (1 mg/kg/hr) during the 6-hour experimental period. Following injury, baseline measurements were taken with PEEP set at the minimum level identified in preliminary experiments to keep the animals alive for 6 hours. In the UI and LPS groups, PEEP was set at 2 cm H_2_O, while in the SW PEEP was set at 6 cm H_2_O. Animals were further randomized into three subgroups (*n *= 8/each): (1) high frequency oscillatory ventilation (HFOV), (2) CMV with the "best" PEEP set according to the minimal respiratory system static elastance (BP-CMV), and (3) CMV with low PEEP (LP-CMV).

In the LP-CMV group, no recruitment manoeuvre (RM) was applied and PEEP was kept at 2 cm H_2_O (in UI and LPS groups) or 6 cm H_2_O (in SW group). In the BP-CMV group, an open lung approach [5] was performed by using a RM, applied as continuous positive airway pressure of 25 cm H_2_O for 40 seconds, followed by a decremental PEEP trial. Initial PEEP was set at 10 cm H_2_O (in UI and LPS groups) or 16 cm H_2_O (in SW group). P_insp _was adjusted to deliver a V_T _of 6 ml/kg body weight. Thereafter, PEEP was reduced in steps of 2 cm H_2_O, and changes in elastance were measured after a 10-minute equilibration period. PEEP was reduced until the elastance of the respiratory system (E_stat,RS_) no longer decreased. PEEP at minimum E_stat,RS _was defined as "best PEEP". Animals were then re-recruited, and "best-PEEP" was applied throughout the experimental period. All other ventilator settings remained unchanged. Airways were not suctioned during the 6 hours of ventilation.

In the HFOV group, the RM and decremental PEEP trial were performed as described for BP-CMV. Once best PEEP was identified, mean airway pressure (P_mean_) at BP-CMV (P_meanBP-CMV_) was recorded. Animals were then switched to HFOV (SensorMedics 3100A; Care Fusion, San Diego, CA, USA) and oscillated at a FiO_2 _of 0.5, an I:E of 1:2 with a frequency of 15 Hz. P_meanHFOV _was set 2 cm H_2_O above P_meanBP-CMV _according to standard recommendations [8]. Pressure amplitude was adjusted to maintain PaCO_2 _within physiological ranges.

At the end of the experiment, a blood gas analysis was performed. To assess respiratory mechanics, the animals were switched back to CMV at the level of PEEP, initially defined as "best PEEP" with P_insp _readjusted to deliver a V_T _of 6 ml/kg body weight for 2 minutes. Respiratory mechanics were then assessed, after which animals were immediately killed.

### Respiratory system mechanics

Tracheal (P_trach_) and oesophageal (P_es_) pressures were recorded during 3 to 4 seconds of airway occlusion at end expiration and end inspiration. E_stat,RS _was computed as E_stat,rs _= ΔP_trach_/V_T_, where ΔP_trach _is the difference between end-inspiratory and end-expiratory tracheal pressure. Static elastance of the chest wall (E_stat,CW_) was computed as ΔP_es_/V_T_, where ΔP_es _is the difference between end-inspiratory and end-expiratory oesophageal pressure. Static lung elastance (E_stat, L_) was calculated as (E_stat,L _= E_stat,RS _- E_stat,CW_).

### Histological examination

At the end of the experiment (6 hours), a laparotomy was done immediately after the determination of lung mechanics (End), and heparin (1,000 IU) was intravenously injected. The trachea was clamped at 5 cm H_2_O PEEP in all groups to standardize pressure conditions. The abdominal aorta and vena cava were sectioned, yielding a massive haemorrhage that quickly killed the animals. Lungs were removed *en bloc*. The right lungs were quick-frozen in nitrogen for mRNA analysis. The left lungs were immersed in 4% formalin and embedded in paraffin. Four-μm-thick slices were cut and haematoxylin and eosin-stained. Morphological examination was performed in a blinded fashion by two investigators using a conventional light microscope at a magnification of ×100 across 10 random, noncoincident microscopic fields. A five-point semiquantitative severity-based scoring system was used as previously described [17]. The pathological findings were graded as negative = 0, slight = 1, moderate = 2, high = 3, and severe = 4. The amount of intra- and extra-alveolar haemorrhage, intra-alveolar oedema, inflammatory infiltration of the interalveolar septa and airspace, atelectasis and overinflation were rated. The scoring variables were added, and a histological total lung injury score per slide was calculated.

### Systemic inflammatory response

To assess the systemic inflammatory response, the concentration of tumour necrosis factor (TNF)-α, interleukin (IL)-1 and IL-6 were measured in blood plasma after the 6-hour experimentation period using the enzyme-linked immunosorbent assay (ELISA) technique according to the manufacturer's instructions (R&D Systems Abingdon, UK). The blood samples were taken immediately before the animals were killed.

### Real-time quantitative PCR

Total mRNA was extracted from the right lungs using TriZOL reagent (Invitrogen GmbH, Karlsruhe, Germany), digested with RNase free DNase I (Invitrogen GmbH) and reverse-transcribed into cDNA using Supersript II Reverse Transcriptase (Invitrogen GmbH) according to manufacturer's instructions. TaqMan™ real-time polymerase chain reaction (RT-PCR) was used for quantitative measurement of mRNA expression of TNF-α, IL-1β, IL-6 and (Pro-) Collagen I (PCI) and III (PCIII) using commercially available primers (TaqMan™ gene expression assay; Applied Biosystems Applera Deutschland GmbH, Darmstadt, Germany: Assay_ID: β-Actin: Rn00667869_m1, TNFα Rn99999017_m1, IL6 Rn99999011_m1, IL1ß Rn00676330_m1, Col1A1 Rn01463848_m1, Col3A1 Rn01437681_m1). All samples were measured in triplicate. Gene expression was normalized to the housekeeping gene b-actin and expressed as fold change relative to control calculated with the ΔΔCT method [18]. To rule out possible differences in relative expression of different housekeeping genes, part of the data was reanalyzed as *post hoc *data using glyceraldehyde 3-phosphate dehydrogenase (GAPDH), leading to comparable results (data not shown).

### Statistical analysis

The normality of the data (Shapiro-Wilk test) and the homogeneity of variances (Levene median test) were tested. In case of physiological data, both conditions were satisfied in all instances and thus two-way ANOVA for repeated measures was used followed by Holm-Sidak's *post hoc *test when required. Physiological data are expressed as means ± SEM. Data from PCR and ELISA analysis (expressed as median (25%-75% quartiles)) were tested using Student's *t*-test or Mann-Whitney rank sum test when appropriate. Ratios (fold changes), indicating the magnitude of response with respect to unventilated controls, were used for PCR analyses. Statistical analyses were performed using SigmaPlot 11.0 (Systat Software GmbH, Erkrath, Germany). The level of significance was set at *P *< 0.05.

## Results

### Effects of saline washout and LPS-induced lung injury at baseline

Following saline washout, PEEP had to be increased from 2 to 6 cm H_2_O as described above. Compared to UI animals, SW injury presented higher P_insp _(12.3 ± 1.4 cm H_2_O *vs*. 26.3 ± 2 cm H_2_O; *P *< 0.001), E_stat,RS _(2.7 ± 0.5 cm H_2_O/ml *vs*. 6.4 ± 1 cm H_2_O/ml; *P *< 0.001), PaCO_2 _(44 ± 7.1 *vs*. 57 ± 8.9 mmHg; *P *< 0.001) and lower PaO_2_/FiO_2 _ratio (P/F, 474 ± 54 mmHg *vs*. 76 ± 18 mmHg; *P *< 0.001).

Compared to UI animals, LPS showed lower P_insp _(12.3 ± 1.4 cm H_2_O *vs*. 10.9 ± 0.8 cm H_2_O; *P *< 0.001) and similar E*stat, RS *(2.7 ± 0.5 cm H_2_O/ml *vs*. 2.9 ± 0.4 cm H_2_O/ml; *P *= 0.092), PaO_2_/FiO_2 _ratio (474 ± 54 mmHg *vs*. 453 ± 59 mmHg; *P *= 0.07), or PaCO_2 _(44.1 ± 7.1 *vs*. 46.3 ± 11.1 mmHg, *P *= 0.939). All baseline values in each UI, SW, and LPS model were comparable (Table 1).

**Table 1:** Baseline parameters

	UI			SW			LPS		
			
	LP-CMV	BP-CMV	HFOV	LP-CMV	BP-CMV	HFOV	LP-CMV	BP-CMV	HFOV
P_insp_	11.8 ± 1.0	12.8 ± 2.0	12.4 ± 0.9	26.1 ± 2.0	25.6 ± 1.3	27.0 ± 2.5	11.2 ± 0.9	10,5 ± 0.8	11.3 ± 0.5
E_stat,RS_	2.7 ± 0.5	2.7 ± 0.5	2.8 ± 0.3	6.4 ± 1.1	6.3 ± 0.7	6.8 ± 1.1	2.9 ± 0.3	2.8 ± 0.4	3.0 ± 0.4
PaO_2_/FiO_2_	504.0 ± 17.4	481.5 ± 44.7	482.8 ± 70.6	73.1 ± 19.9	76.8 ± 17.9	78.0 ± 13.7	477.2 ± 48.6	428.5 ± 80.1	458.4 ± 33.3
PaCO_2_	46.2 ± 5.3	39.2 ± 8.0	46.8 ± 5.7	56.6 ± 7.2	63.0 ± 7.2	53.8 ± 10.1	45.0 ± 8.2	44.7 ± 15.7	49.0 ± 8.1

UI, uninjured; SW, lung injury induced by saline washout; LPS, lung injury induced by intraperitoneal/intravenous E. coli lipopolysaccharide; BL, baseline measurements; LP-CMV, controlled mechanical ventilation with low PEEP; BP-CMV, controlled mechanical ventilation with "best" PEEP; HFOV, high frequency oscillatory ventilation; Pinsp, End-inspiratory plateau pressures at baseline; Estat, RS, Respiratory system elastance (Estat, RS) at baseline; PaO_2_/FiO_2_, PaO_2_/FiO_2 _index at baseline; PaCO_2_, PaCO_2 _at baseline. Values are means ± standard deviation. No significant differences were noted in the respective treatment groups at baseline. UI, uninjured; SW, lung injury induced by saline washout; LPS, lung injury induced by intraperitoneal/intravenous *E. coli *lipopolysaccharide.

**Figure 2:**
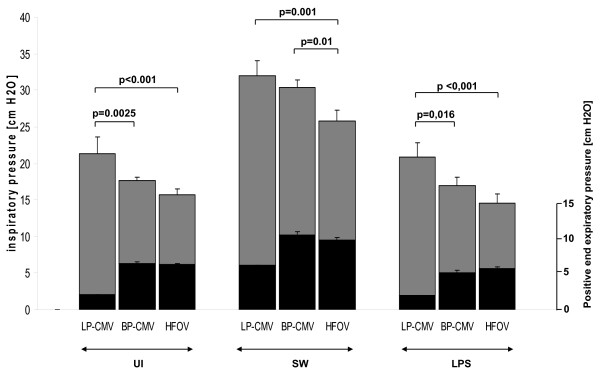
**End-inspiratory plateau pressures after 6 hours of mechanical ventilation**. Black bars represent the level of PEEP. Values are means ± SEM of eight animals in each group.

Best PEEP was set at 6.2 ± 0.5 cm H_2_O in the UI group, 9.9 ± 1.1 cm H_2_O in the SW group (*P *< 0.001 *vs*. UI group) and 5.3 ± 1 cm H_2_O (*P *= 0.01 *vs*. UI group) in the LPS group (Figure 2).

### Effects of LP-CMV, BP-CMV and HFOV

#### Respiratory system mechanics

**Figure 3:**
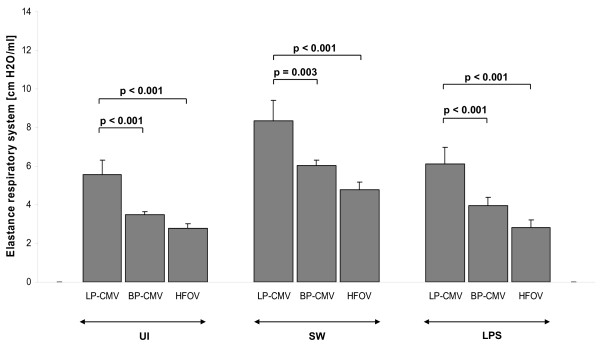
**Respiratory system elastance (E_stat,RS_) after 6 hours of mechanical ventilation**. Values are means ± SEM of eight animals in each group.

After 6 hours in all groups, P_insp _was higher in the LP-CMV compared to HFOV (Figure 2). E_stat,RS _increased with time in LP-CMV in all groups. Additionally, with HFOV, E_stat,RS _decreased with time in SW, while in LPS E_stat,RS _increased with BP-CMV (Figure 3). All changes in respiratory system mechanics observed within the three main groups were attributable to changes in lung mechanics, as E_stat,CW _did not change.

#### Gas exchange

**Figure 4:**
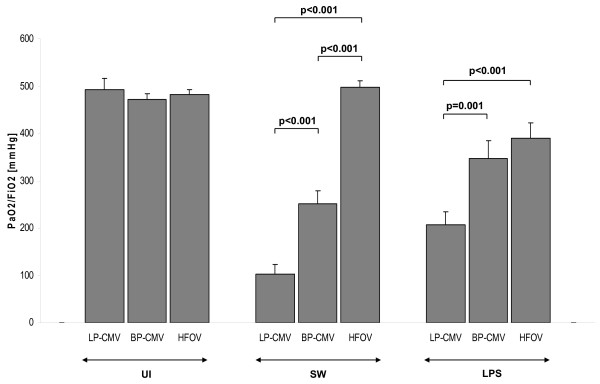
**PaO_2_/FiO_2 _index after 6 hours of mechanical ventilation**. Values are means ± SEM of eight animals in each group.

**Figure 5:**
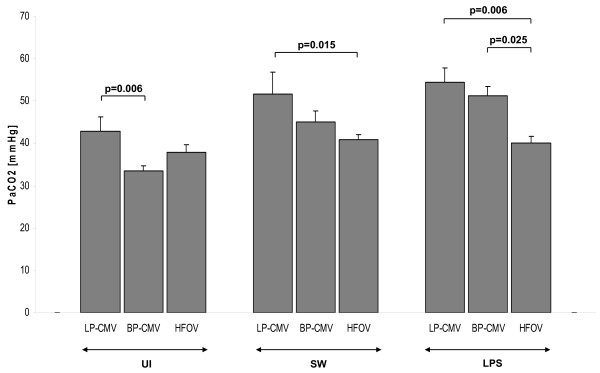
**PaCO_2 _after 6 hours of mechanical ventilation**. Values are means ± SEM of eight animals in each group.

In UI animals, no major effects of the ventilation modes were observed on PaO_2_/FiO_2 _ratio (Figure 4), but PaCO_2 _was more reduced in BP-CMV (33.5 ± 1.1 mmHg) than in LP-CMV (42.91 ± 3.4 mmHg) at 6 hours (*P *= 0.006) (Figure 5). Compared to baseline, HFOV also improved ventilation (PaCO_2_: 46.8 ± 2 *vs*. 37.9 ± 1.8 mmHg; *P *= 0.007).

In SW animals, BP-CMV and HFOV presented a greater PaO_2_/FiO_2 _ratio at end compared to baseline (*P *< 0.001). The increase in PaO_2_/FiO_2 _ratio after 6 hours of HFOV was more pronounced than that of BP-CMV (497.8 ± 13.8 *vs*. 250.8 ± 28.1 mmHg; *P *< 0.001) (Figure 4). PaCO_2 _decreased after 6 hours of HFOV compared to baseline and LP-CMV (Figure 5). In LPS, there was a deterioration in PaO_2_/FiO_2 _ratio in LP-CMV and BP-CMV groups, which was more pronounced in LP-CMV (*P *= 0.001). Six hours of LP-CMV impaired ventilation (45 ± 3.1 *vs*. 54.5 ± 3.4 mmHg; *P *= 0.017). Conversely, HFOV reduced PaCO_2 _(48.9 ± 2.9 *vs*. 40.1 ± 1.7 mmHg; *P *= 0.02) with no significant change in PaO_2_/FiO_2 _ratio.

#### Histological examination

**Figure 6:**
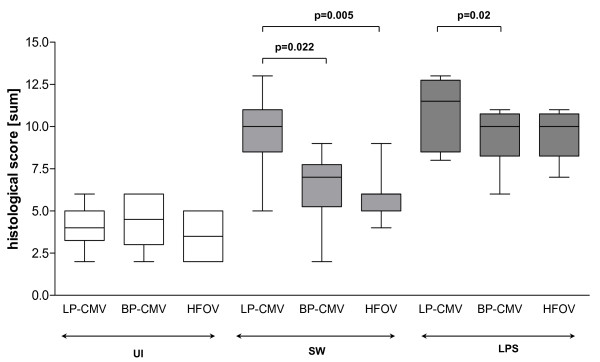
**Histological total lung injury score**. Boxes show interquartile (25%-75%) range, whiskers encompass range and horizontal lines represent median value.

As shown in Figure 6, the histological total lung injury score was higher in SW and LPS compared to UI. In UI, ventilatory mode did not affect the histological total lung injury score. In SW, the total lung injury score was higher for LP-CMV compared to both BP-CMV and HFOV. Following LPS injury, the total lung injury score was higher in LP-CMV compared to BP-CMV.

In UI, LP-CMV induced more atelectasis (Table 2). In SW, LP-CMV yielded higher oedema. In LPS, all ventilatory strategies led to higher inflammation compared to UI and SW. Inflammation and atelectasis were also more intense in LP-CMV than in BP-CMV and HFOV (Table 2).

**Table 2:** Histological lung injury score

		UI	SW	LPS
Haemorrhage	LP-CMV	0.0 (0.0/0.0)	0.0 (0.0/0.0)	2.0 (1.0/3.0)
	BP-CMV	0.0 (0.0/0.0)	1.0 (0.0/1.0)	1.0 (1.0/3.0)
	HFOV	0.0 (0.0/0.0)	1.0 (0.0/1.5)	2.0 (1.0/2.0)
				
Inflammation	LP-CMV	1.0 (0.0/2.0)	2.0 (2.0/3.0)	4.0 (4.0/4.0)^a,b^
	BP-CMV	1.0 (0.75/1.0)	1.0 (1.0/2.0)	3.0 (3.0/4.0)
	HFOV	1.0 (0.0/1.25)	2.0 (1.0/2.0)	3.0 (3.0/4.0)
				
Oedema	LP-CMV	0.0 (0.0/0.0)	3.0 (2.5/4.0)^a,b^	2.0 (1.0/2.0)
	BP-CMV	0.0 (0.0/0.0)	0.0 (0.0/2.0)	2.0 (0.0/2.0)
	HFOV	0.0 (0.0/0.0)	0.0 (0.0/0.0)	1.0 (0.0/2.0)
				
Atelectasis	LP-CMV	2.5 (1.5/3.25)^b^	2.0 (1.0/2.0)	2.5 (2.0/3.75)^a^
	BP-CMV	1.0 (1.0/2.0)^c^	2.0 (1.0/2.5)	1.0 (0.0/2.0)
	HFOV	0.0 (0.0/1.0)	1.0 (0.5/1.5)	1.0 (1.0/2.0)
				
Overinflation	LP-CMV	0.0 (0.0/1.5)^a,b^	2.0 (2.0/3.0)	1.0 (0.25/1.75)
	BP-CMV	2.0 (1.75/3.25)	1.0 (1.0/2.0)	2.0 (1.0/2.0)
	HFOV	2.0 (1.75/2.5)	2.0 (1.5/3.5)	3.0 (1.0/3.0)
				
Total lung injury score (sum)	LP-CMV	4.0 (3.75/5.0)	10.0 (9.0/11.0)^a,b^	12 (10.25/13.0)^a^
	BP-CMV	4.5 (3.0/6.0)	7.0 (5.5/7.5)	10.0 (8.0/10.0)
	HFOV	3.5 (2.0/5.0)	6.0 (5.0/6.0)	10.0 (9.0/11.0)

Baseline measurements; LP-CMV, controlled mechanical with low PEEP; BP-CMV, controlled mechanical ventilation with "best" PEEP; HFOV, high-frequency oscillatory ventilation. Values are medians and interquartile (25%-75%) range. ^a^*P *< 0.05 LP-CMV *vs*. BP-CMV. ^b^*P *< 0.05 LP-CMV *vs*. HFOV. ^c^*P *< 0.05 BP-CMV *vs*. HFOV.

#### Lung tissue inflammatory response

No differences in lung tissue inflammatory response were observed with the use of different ventilatory modes in UI animals. In SW animals ventilated with low PEEP, IL-1β and IL-6 expression was higher compared to BP-CMV and HFOV, respectively. IL-6 mRNA expression was also increased in LPS animals ventilated with LP-CMV compared to both open lung strategies (Table 3). In LPS-injured lungs, HFOV caused less TNF-α expression than BP-CMV.

**Table 3:** Lung inflammatory and fibrotic response

		UI	SW	LPS
TNF-α	LP-CMV	5.9 (4.6/7.8)	2.3 (1.9/2.9)	13.1 (12.2/15.5)
	BP-CMV	6.0 (4.9/7.4)	2.8 (2.9/3.0)	21.1 (12.8/24.1)^c^
	HFOV	6.5 (3.7/7.4)	3.5 (1.8/4.0)	12.7 (11.6/15.6)
				
Interleukin-1β	LP-CMV	6.4 (4.6/7.5)	2.9 (2.4/6.1)^a,b^	8.4 (7.6/9.9)
	BP-CMV	4.5 (3.5/6.6)	2.2 (1.5/3.0)*	10.4 (8.3/11.1
	HFOV	4.3 (3.7/5.9)	2.2 (1.8/3.2)*	9.5 (8.4/11.2)
				
Interleukin-6	LP-CMV	24.0 (10.2/30.5)	625.2 (399.8/880.0)^a,b^	1278.5 (1187.4/1390.2)^a,b^
	BP-CMV	16.0 (6.7/23.7)	380.7 (205.4/417.5)	498.4 (381.2/568.2)
	HFOV	5.7 (3.6/14.7)	367.4 (182.9/496.1)	446.1 (252.6/563.8)
				
Procollagen I	LP-CMV	1.0 (0.8/1.2)*^a^	0.5 (0.6/0.8)*	0.4 (0.3/0.4)
	BP-CMV	1.4 (1.1/2.0)^c^	0.6 (0.5/1.2)*	0.2 (0.1/0.4)
	HFOV*	1.0 (0.7/1.5)*	0.8 (0.4/1.0)*	0.3 (0.3/0.5)
				
Procollagen III	LP-CMV	0.5 (0.5/0.6)	0.3 (0.2/0.4)	0.2 (0.1/0.2)
	BP-CMV	0.6 (0.5/0.8)	0.3 (0.2/0.3)	0.2 (0.1/0.3)
	HFOV	0.5 (0.4/0.6)	0.3 (0.2/0.3)	0.2 (0.2/0.3)

UI, uninjured; SW, lung injury induced by saline washout; LPS, lung injury induced by intraperitoneal/intravenous *E. coli *lipopolysaccharide; LP-CMV, controlled mechanical ventilation with low PEEP; BP-CMV, controlled mechanical ventilation with "best" PEEP; HFOV, high frequency oscillatory ventilation. Values are medians and interquartile (25%-75%) range and are presented as fold changes relative to unventilated control group. *P > 0.05 *vs*. unventilated control group. ^a^*P *< 0.05 LP-CMV *vs*. BP-CMV. ^b^*P *< 0.05 LP-CMV *vs*. HFOV. ^c^*P *< 0.05 BP-CMV *vs*. HFOV.

#### Procollagen expression

In UI, PCI mRNA expression in lung tissue was higher in BP-CMV compared to HFOV and LP-CMV, while no differences were observed in the SW animals (Table 3). LPS injury induced a substantial and uniform decrease in PCI mRNA expression.

PCIII mRNA expression was significantly and uniformly lower throughout all groups and modes of MV compared to unventilated controls, with the reduction being most pronounced following LPS (Table 3).

#### Systemic inflammatory response

The systemic inflammatory response elicited by 6 hours of ventilation of uninjured lungs was lower for HFOV compared to both LP-CMV and BP-CMV (Table 4). In SW animals ventilated with low PEEP, systemic IL-6 levels were higher compared to BP-CMV.

**Table 4:** Systemic inflammatory response

	Control		UI	SW	LPS
TNF-α (pg/ml)	0 (0/0)	LP-CMV	18.0 (15.0/29.0)	0 (0/17.75)*	52.0 (45.0/136.5)
		BP-CMV	49.0 (20.0/55.5)^c^	0 (0/0)*	34.0 (26.25/48.75)
		HFOV	9.0 (9.0/16.5)	0 (0/0)*	29.5 (27.0/39.25)
					
Interleukin-1β (pg/ml)	19.5 (15.5/24.25)	LP-CMV	17.5 (15.0/24.25)^b^	11.5 (0/26.25)	51.0 (47.0/117.0)^b^
		BP-CMV	16.5 (15.0/34.5)^c^	0 (0/5.5)	46.0 (36.0/265.25)^c^
		HFOV	0 (0/0)	6 (0/6.25)	31.5 (23.5/33.25)
					
Interleukin-6 (pg/ml)	41.0 (34.0/48.0)	LP-CMV	232.0 (187.0/290.0)^b^	185.0 (149.0/218.0)^a^	22320.0 (16375.0/65440.0)
		BP-CMV	262.0 (232.0/276.0)^c^	53.0 (40.0/127.5)	14395.0 (9300.0/54967.5)
		HFOV	78.0 (55.0/145.5)	169.0 (95.5/239.0)	35930.0 (20090.0/43025.0)

UI, uninjured; SW, lung injury induced by saline washout; LPS, lung injury induced by intraperitoneal/intravenous *E. coli *lipopolysaccharide; BL, baseline measurements; LP-CMV, controlled mechanical ventilation with low PEEP; BP-CMV, controlled mechanical ventilation with "best" PEEP; HFOV, high frequency oscillatory ventilation. Values are medians and interquartile (25%-75%) range. ^a^*P *< 0.05 LP-CMV *vs*. BP-CMV. ^b^*P *< 0.05 LP-CMV *vs*. HFOV. ^c^*P *< 0.05 BP-CMV vs. HFOV.

Following 6 hours of intravenous infusion of LPS, a uniform massive inflammatory response was observed with only very minor differences in IL-1β favouring HFOV. This massive inflammatory response was also reflected by higher dose requirements of norepinephrine and additional fluid to maintain a systolic blood pressure above 60 mmHg compared to SW and UI groups. There were no differences within groups for fluid and norepinephrine requirements, respectively.

## Discussion

In the present study, we investigated the effects of "open lung" strategies using HFOV or CMV (BP-CMV) compared to low-PEEP CMV (LP-CMV) on gas-exchange, hemodynamic, respiratory system static elastance, pulmonary histology, cytokines and types I and III procollagen (PCI and PCIII) mRNA expression in lung tissue as well as plasma cytokines following 6 hours of mechanical ventilation. We found that (1) in the UI group, BP-CMV and HFOV compared to LP-CMV did not provide major benefits except for maintaining respiratory system static elastance; (2) in both SW and LPS groups, HFOV and BP-CMV improved gas exchange and mechanics with lower lung injury scores compared to LP-CMV; (3) in the SW group, HFOV yielded better oxygenation than BP-CMV; (4) in SW group, IL-6 mRNA expression was lower during BP-CMV or HFOV compared to LP-CMV, while in the LPS group inflammatory response remained largely independent of ventilatory mode; and (5) PCIII mRNA expression decreased in all groups and ventilatory modes, mainly in the LPS model.

We observed that "open lung" ventilatory strategies using HFOV or CMV improved respiratory function and minimized lung injury compared to LP-CMV. Setting P_meanHFOV _2 cm H_2_O above the P_meanBP-CMV _following a recruitment manoeuvre is as beneficial as BP-CMV. Both open lung strategies were able to reduce the biotrauma as assessed by pulmonary IL-6 expression compared to LP-CMV. The fact that no major differences in IL-6 expression during HFOV and BP-CMV were observed suggests the limited ability of HFOV to minimize biotrauma compared to optimized conventional ventilatory approaches. To assess the effects of the underlying lung injury model, ventilatory strategies were tested in three different situations: without injury and following SW and LPS lung injury. We tested uninjured animals because the effects of "open lung" strategies during general anaesthesia and paralysis in healthy lungs are a matter of debate [19]. The SW model was chosen because it provides an ideal way to test the effects of different ventilatory strategies on the development of tissue injury. In fact, tissue injury results more from the ventilatory strategy than from the saline lavage, as surfactant depletion facilitates alveolar collapse and increases the likelihood of mechanical injury to the alveolar walls during repeated cycles of opening/closing unless optimum PEEP is applied [20]. Also, SW profoundly affects lung mechanics and gas exchange [16,21]. The LPS model was selected because it mimics a situation of sepsis [22] and because it is characterized by direct endothelial insult, but without significant impact on lung mechanics [23]. We used a two-hit model with LPS applied intraperitoneally 24 hours before and by continuous intravenous infusion throughout the experimental period, resulting in a massive systemic and pulmonary inflammatory response as well as high histological injury scores.

The "best" PEEP during CMV was set according to the lower static elastance of the respiratory system during a decremental PEEP trial following a RM. Differently from gas exchange, lung mechanics are not affected by changes in regional perfusion [24] and are mainly determined by changes in pulmonary structure [25]. In addition, RM has been found effective to optimize recruitment before PEEP application [26].

The expression of different inflammatory and fibrogenic mediators in the lung tissue was measured. PCI and PCIII mRNA expressions were analyzed to better understand the different moments of fibrogenesis. Type III collagen fibre is more flexible and susceptible to breakdown and predominates early in the course of lung injury, whereas type I collagen (composed of thicker and cross-linked fibrils) is more prevalent in the late phase [27].

The effects of different ventilatory strategies are discussed individually for each of the three groups, since it was not the aim of our study to compare modes of ventilation across injury models.

### Uninjured animals

In uninjured animals, LP-CMV compared to BP-CMV and HFOV maintained gas exchange and increased static elastance of the respiratory system but did not promote lung injury. As expected, we observed a high amount of atelectasis in LP-CMV and none in BP-CMV and HFOV. Conversely, more overinflation was seen in BP-CMV and HFOV. Increased atelectasis may explain the rise in static elastance after 6 hours of mechanical ventilation. Although PEEP between 3 to 5 cm H_2_O associated with low tidal volume has been used as a lung-protective strategy, we found significant differences between PEEP 2 cm H_2_O (low PEEP) and 6 cm H_2_O (best PEEP) regarding mechanics and atelectasis. This may be due to the fact that the experimental period in our study was prolonged to 6 hours compared to 1 hour in most comparable studies.

Overall lung inflammatory response, as assessed by mRNA expression of TNF-α, IL-1β and IL-6, showed no major differences among ventilatory modes. PCI mRNA expression in lung tissue increased in BP-CMV compared to LP-CMV, HFOV and unventilated controls. On the other hand, PCIII mRNA expression uniformly decreased after 6 hours in LP-CMV, BP-CMV or HFOV. PCIII mRNA expression has been reported to be an early marker of lung parenchyma remodelling [14,17,28,29] and shown to be higher in lungs subjected to elevated airway pressures [14,17], high inflation [30] or cyclic mechanical strain [29]. In a recent study, Santana and co-workers [31] showed unchanged PCIII mRNA expression in lung tissue following 1 hour of MV at low V_T _and zero end-expiratory pressure in uninjured rat lungs. Our study is the first to report decreased PCIII mRNA expression after 6 hours of low V_T _ventilation using low or "best" PEEP. Conversely, we observed increased levels of PCI mRNA in animals ventilated with BP-CMV, which may be attributed to increased pulmonary stress and strain [31]. These differences may be related to (1) the different experimental time period (6 hours in the present study *vs*. 1 hour in previous studies) [14,17,28-32] and (2) the use of low PEEP level.

### Saline lavage and LPS-induced lung injury

These two models induced major functional and histological differences. SW was characterized by higher static elastance of the respiratory system, oedema and overinflation, with major deterioration in gas exchange. It cannot be ruled out that part of the oedema observed histologically in the SW animals was associated with saline not reabsorbed or removed during the lavage process. As baseline parameters were comparable for all SW animals, we can speculate that this potential error does not interfere with the validity of the results. Compared to SW, LPS was characterized by more inflammation, haemorrhage and atelectasis. In both ALI models, LP-CMV compared to BP-CMV and HFOV resulted in deterioration of gas exchange, respiratory system mechanics and lung histology.

Our data suggest that LP-CMV-induced injury is different in relation to initial damage as compared to BP-CMV and HFOV: higher leakage and alveolar oedema in SW [32], with less epithelial-endothelial damage with migration of neutrophils, inflammation and marked interstitial oedema with atelectasis formation in LPS [20]. LPS promotes systemic inflammation that could prime alveolar macrophages *a posteriori*, but initially LPS does not cause severe endothelial/epithelial damage [23].

On the other hand, HFOV, but not BP-CMV, was able to maintain oxygenation and lung mechanics in LPS, which suggests a potential role for HFOV early in the course of severe sepsis. These beneficial effects are probably related to the higher mean airway pressures during HFOV. It should be noted, however, that these increased airway pressures did not cause haemodynamic compromise or increased fluid or norepinephrine requirements in HFOV animals compared to BP-CMV or LP-CMV. The increased shear stress and strain imposed by opening and stretching of collapsed lung in the dependent regions due to insufficient levels of PEEP was probably high enough to stimulate an increased parenchymal inflammatory response as assessed by elevated levels of IL-6 mRNA expression in the lung tissue. IL-6, differently from TNF-α or IL-1β, is expressed and released during several hours and may therefore be more suitable to assess the effects of the ventilatory strategies used [33].

After 6 hours of MV and PCIII, but not PCI, mRNA expression was significantly and uniformly lower compared to unventilated controls independent of ventilatory mode in the ALI experimental model. This finding is in contrast to previous studies mostly showing that short-term (1 hour) "lung-protective" MV does not alter PCIII mRNA expression in lung tissue in different experimental models of ALI, while less protective modes result in increased PCIII mRNA expression [17,28-31]. It is likely that time can play a relevant role in determining different activation in collagen response; however, we are not aware of any previous study investigating the kinetics of collagen formation within the first 6 hours after ALI. We could speculate that inflammation, and probably IL-6, may promote a reduction in collagen synthesis [34]. Further studies are required to address these issues.

The systemic inflammatory response was higher in LPS compared to SW [21,23] but was unaffected by different ventilatory modes, as previously reported [35,36]. Thus, our data suggest that the local, and not the systemic, inflammatory response should be taken into account when evaluating lung injury induced by different ventilatory treatments. Furthermore, the systemic response appears to be more correlated with factors that are external to the lung.

### Limitations

The current study has several limitations that need to be addressed. First, PEEP levels were not identical in the three modes across groups, since low PEEP had to be set at 6 cm H_2_O in SW compared to 2 cm H_2_O in UI and LPS in LP-CMV animals to prevent detrimental hypoxemia. Likewise, PEEP levels (BP-CMV) and P_meanBP-CMV _(HFOV) were different between groups as they were individually titrated. However, the primary aim of the study was to compare three defined ventilatory strategies in different lung conditions rather than compare the individual strategies between groups. Second, we studied three ventilatory strategies in different lung conditions: uninjured lungs, animals undergoing acute saline lung lavage as well as animals exposed to LPS already 24 hours prior to the experiment as part of a "double-hit" injury. This "double hit" approach was used to get a stable and reproducible LPS model. Therefore, this study comprises data on the effects of different ventilatory strategies not only for different types of injury but also for noncomparable injury "times". Our aim was to assess the effectiveness of open-lung ventilatory strategies over a wide range of lung conditions rather than compare theses different lung conditions. Therefore, the different timing of injury does not necessarily interfere with a proper analysis of the results.

Third, additional fluid was given to LPS animals to maintain haemodynamics. While this may promote oedema formation, fluid administration is a key element of resuscitation in septic shock. As identical amounts of fluid were given to each LPS animal, the interpretation of data within the LPS group was not affected.

Fourth, some methodological issues regarding the method used for RNA quantification of collagen turnover in this study compared to other studies deserve to be mentioned. Assuming that differences in the overall collagen RNA turnover in the different treatment groups during our short observation period might be rather low, we utilized the TaqMan™ PCR system for real-time RNA quantification, which is considered one of the best quantitative [37] and most sensitive [38] approaches for measurement of RNA currently available. In contrast to most studies so far, we used the housekeeping b-actin gene rather than GAPDH as reference gene, but this did not interfere with our results.

## Conclusions

In different animal ALI models, open lung ventilatory strategies using HFOV or CMV improved respiratory function and minimized lung injury compared to LP-CMV. Open lung HFOV with P_meanHFOV _set 2 cmH_2_O above the P_meanBP-CMV _following a recruitment manoeuvre is as beneficial as BP-CMV.

## Key messages

• Open lung ventilatory strategies associated with HFOV or best PEEP (BP)-CMV improved respiratory function and minimized lung injury more than low PEEP (LP)-CMV.

• HFOV with P_meanHFOV _set 2 cm H_2_O above the P_meanBP-CMV _following a recruitment manoeuvre was as beneficial as BP-CMV.

• After 6 hours of protective ventilation, PCIII mRNA expression was significantly and uniformly lower compared to unventilated controls in all groups and modes of MV.

## Abbreviations

ALI: acute lung injury; ARDS: adult respiratory distress syndrome; BP: best PEEP; E_stat_,_RS_: static respiratory system elastance; E_stat,l_: static lung elastance; E_stat,cw_: static chest wall elastance; IL: interleukin; LP: low PEEP; LPS: lipopolysaccharide; PC: procollagen; PCR: polymerase chain reaction; PEEP: positive end-expiratory pressure; P_es_: oesophageal pressure; P_plat_: inspiratory plateau pressure; RM: recruitment manoeuvre; SW: saline washout; V_T_: tidal volume; ZEEP: zero end-expiratory pressure.

## Competing interests

The authors declare that they have no competing interests.

## Authors' contributions

JK, PP and TL participated in the study design. JK, CT, LZ, BY and TL performed the study. JK, PP, BY and TL processed the data and performed the statistical analysis. JK, PP, PRMR and TL wrote the manuscript. All authors revised the manuscript and approved its final version.
